# Estrogen and progesterone-related gene variants and colorectal cancer risk in women

**DOI:** 10.1186/1471-2350-12-78

**Published:** 2011-05-31

**Authors:** Jennifer H Lin, JoAnn E Manson, Peter Kraft, Barbara B Cochrane, Marc J Gunter, Rowan T Chlebowski, Shumin M Zhang

**Affiliations:** 1 Division of Preventive Medicine, Brigham and Women's Hospital and Harvard Medical School, Boston, MA, USA; 2 Department of Epidemiology, Harvard School of Public Health, Boston, MA, USA; 3 de Tornyay Center for Healthy Aging, University of Washington, Seattle, WA, USA; 4 Department of Epidemiology and Population Health, Albert Einstein College of Medicine Yeshiva University, Bronx, NY, USA; 5 Department of Medicine, Los Angeles Biomedical Research Institute at Harbor-UCLA Medical Center, Torrance, CA, USA

## Abstract

**Background:**

Observational studies and randomized trials have suggested that estrogens and/or progesterone may lower the risk for colorectal cancer. Inherited variation in the sex-hormone genes may be one mechanism by which sex hormones affect colorectal cancer, although data are limited.

**Method:**

We conducted a comprehensive evaluation of single nucleotide polymorphisms (SNPs) in genes encoding 3 hormone receptors (*ESR1, ESR2, PGR*) and 5 hormone synthesizers (*CYP19A1 and CYP17A1, HSD17B1, HSD17B2, HSD17B4*) among 427 women with incident colorectal cancer and 871 matched controls who were Caucasians of European ancestry from 93676 postmenopausal women enrolled in the Women's Health Initiative Observational cohort. A total of 242 haplotype-tagging and functional SNPs in the 8 genes were included for analysis. Unconditional logistic regression with adjustment for age and hysterectomy status was used to estimate odds ratios (ORs) and 95% confidence intervals (CIs).

**Results:**

We observed a weak association between the *CYP17A1 *rs17724534 SNP and colorectal cancer risk (OR per risk allele (A) = 1.39, 95% CI = 1.09-1.78, corrected p-value = 0.07). In addition, a suggestive interaction between rs17724534 and rs10883782 in 2 discrete LD blocks of *CYP17A1 *was observed in relation to colorectal cancer (empirical p value = 0.04). Moreover, one haplotype block of *CYP19A1 *was associated with colorectal cancer (corrected global p value = 0.02), which likely reflected the association with the tagging SNP, rs1902584, in the block.

**Conclusion:**

Our findings offer some support for a suggestive association of *CYP17A1 *and *CYP19A1 *variants with colorectal cancer risk.

## Background

Epidemiologic studies have consistently shown that an increase in female hormones such as estrogens and progestin as a result of pregnancy or use of exogenous steroid hormones is associated with a lower risk for developing colorectal cancer [1-3]. In support of these findings, the Women's Health Initiative (WHI) estrogen plus progestin (E+P) clinical trial reported a 40% lower risk for colorectal cancer in the treatment group compared with the placebo group [4,5]. By contrast, the other WHI estrogen-alone (E-alone) trial among hysterectomized women did not find a lower risk of colorectal cancer in the treatment group [6]. Two recent observational studies also reported no reduced risk for colorectal cancer incidence among postmenopausal women with higher circulating levels of estradiol and estrone [7,8]. Findings from these studies seemingly suggest that progesterone, but not estrogen, may be the key candidate for risk reduction in colorectal cancer. Alternatively, the risk associated with sex hormones may be under genetic control, as these hormones bind to their respective receptors to exert biological actions in target tissues such as the colorectum. Genes responsible for sex-hormone synthesis and metabolism also affect changes in sex hormone concentrations, and variation in these genes may affect risk for disease development.

Few candidate-gene studies have evaluated variation in sex-hormone genes in relation to colorectal cancer risk and findings have been mixed. Some [9,10] but not all [11,12] studies reported a potential link between genetic variation in estrogen receptors and colorectal cancer development. To date, at least 3 phase-design genome-wide association scan (GWAS) studies of colorectal cancer have been undertaken, which identified several novel susceptibility loci mapping to 1q41, 3q26.2, 8q23.3, 8q24, 10p14, 12q13.13, 14q22.2, 15q13, 16q22.1, 18q21, 19q13.1, 20p12.3, and 20q13.33 [13-20]. However, none of these detected regions harbor genes involved in sex hormone synthesis or actions. Two of the nearest sex-hormone genes, HSD17B2 (16q24.1) and CYP19A1 (15q21.1), are at least 13 million basepairs (bp) distant from the GWAS loci. These observations suggest that the individual effect of hormone-related genes on colorectal cancer risk is not large enough to be detected at the genome-wide significance level (ie, p value <10^-7 ^to 10^-8^). Although the association of colorectal cancer with sex hormone genes may be weak at an individual level, the overall association may be enhanced if the contributions attributable to individual loci are combined [21,22]. In addition, the association of colorectal cancer risk with sex hormone genes may also be affected by potential modifiers such as hormone therapy (HT) use [10,23] and BMI [24].

In this case-control study nested in a large cohort of postmenopausal women, we undertook a comprehensive evaluation of common and putative functional variants in the genes encoding estrogen and progesterone receptors (*ESR1, ESR2, PG*R) and enzymes responsible for critical steps in the conversion of progesterone or androgens to estrogens (*CYP19A1 and CYP17A1*) and in the formation of active estrogens (*HSD17B1, HSD17B2, HSD17B4*) in relation to colorectal cancer risk. We additionally tested the combined effects of multiple loci on colorectal cancer risk and evaluated effect modification by several risk modifiers on the association between sex hormone genes and disease risk.

## Methods

### Study population

We conducted a case-control study nested in the Women's Health Initiative Observational cohort (WHI-OS), a large, multifaceted study designed to advance our understanding of the determinants of major chronic diseases in 93,676 postmenopausal women aged 50 to 79 years who were recruited at 40 different clinical centers across the United States between October 1, 1993 and December 31, 1998 [25]. At baseline, women provided informed consent and completed questionnaires regarding demographic and behavioral factors, medical history, and use of medications including HT. A physical examination was conducted that included measurements of height and weight and circumferences of the waist and hip. Blood samples were obtained following an overnight fast of at least 8 hours, and were immediately centrifuged and stored at -70°C.

Each year, participants were asked whether they had been newly diagnosed with colorectal cancer. Case status and detailed diagnosis were then confirmed through centralized review of all pathology reports, discharge and consultant summaries, operative and radiology reports, and tumor registry abstracts. As of September 12 2005, 472 women of European ancestry were identified with a confirmed diagnosis of colorectal cancer. Controls were matched to cases with a ratio of 2:1 frequency on age at screening for participation in the WHI-OS at baseline, ethnicity, hysterectomy status, and prevalent conditions at baseline. A total of 24 (9 cases and 15 controls) with insufficient or lack of DNA were subsequently excluded from the analysis. We additionally excluded 16 women who had reported a cancer history at baseline, which yielded a total of 460 cases and 916 controls in the present analysis.

In our exploratory analysis, we examined whether the potential association of sex hormone genes and colorectal cancer might be generalized to other ethnic groups. We included 58 African Americans with a confirmed diagnosis of colorectal cancer and 116 matched controls, all of whom were free of cancer history at baseline, from the WHI-OS for the analysis.

Written informed consent was obtained from all participants in this study, and the study protocols were reviewed and approved by the Brigham and Women's Hospital Institutional Review Board.

### Single nucleotide polymorphism (SNP) selection

We first selected a set of tagging SNPs that capture common variation and linkage disequilibrium (LD) structure across each of the 8 hormone genes based on the studies from the Breast and Prostate Cohort Consortium project (BPC3), which has performed SNP discovery and dense genotyping to capture most common haplotype diversity in 70 American Caucasians (http://www.uscnorris.com/MECGenetics). The tagging SNPs, chosen from common SNPs with a minor allele frequency (MAF) of 1% or greater among whites, predicted an *R*_h_^2 ^of 0.70 or greater between observed haplotypes and those predicted based on tagging SNP genotypes [26-30]. Altogether, 179 SNPs were selected from the 8 genes.

We next undertook a literature search to identify putative functional SNPs in the selected genes that had been associated with risk of cancers including colorectal cancer. Most of these chosen SNPs were either at coding (synonymous or nonsynonymous) or promoter regions. We identified a total of 17 SNPs from the *CYP19A1, HSD17B4, ESR1, ESR2*, and *PGR *genes.

To further enrich the gene density, we used the Tagger program implemented in Haploview software [31] to identify additional SNPs within the gene regions as well as 10-20kb upstream and downstream of each gene and forced in both the tagging SNPs identified by the BPC3 studies and the functional SNPs selected from the literature. The data source for tagging SNP selection was from the CEPH Utah residents with European ancestry in the International Hapmap Project on the National Center for Biotechnology Information Build 35 assembly available in July 2006 (http://www.hapmap.org). Selection of tagging SNPs was based on a pairwise correlation coefficient (r^2^) of 0.7 or greater between tagging SNPs and untyped SNPs and a MAF of 5% or greater in the CEPH population. As a result, we selected additional 76 tagging SNPs from the 8 genes (Table 1), yielding a total of 272 SNPs for subsequent genotyping.

**Table 1:** Characteristics of the selected sex-steroid synthesizing enzymes and receptors

Gene	Chromosome	Covered region (in bp)	**# of tested SNPs**^**1**^	**LD blocks**^**2**^
CYP17A1	10q24.3	104562266-104595511	11	2
CYP19A1	15q21.1	49269214-49434085	45	5
HSD17B1	17q11-q21	37943139-37970046	6	2
HSD17B2	16q24.1-q24.2	80608616-80698062	16	4
HSD17B4	5q21	118797197-118889612	7	2
ESR1	6q25.1	151999305-152477851	121	22
ESR2	14q23.2	63750946-63838055	11	2
PGR	11q22-q23	100396881-100529492	25	4

^1^. SNPs with Hardy-Weinberg equilibrium and a minor allele frequency of ≥5% in the Women's Health Initiative Observational Cohort (WHI-OS) study Caucasians.

^2^. From the WHI-OS Caucasian controls.

### Genotyping

DNA was extracted from the buffy coat fraction of centrifuged blood using the QIAmp Blood Kit (Qiagen, Chatsworth, CA). Genotyping determination was performed with the Sequenom MassARRAY Genotyping system at the Partners Genotyping Facility (Boston, MA). Briefly, a multiplexed PCR and then a minisequencing reaction were performed in a single well. The size of reaction products was determined directly by MALDI-TOF mass spectrometry, yielding genotype information. Laboratory personnel were blinded to case-control status. Quality control (QC) of Sequenom Genotyping was carried out by repeating the genotyping on 66 duplicate samples with an average concordance rate of 99.7% in all typed SNPs. The average genotyping drop-out rates for all SNPs were 4.3%, ranged from 0.6% to 17.5%.

### Statistical Analysis

Genotype quality and filtering were first performed using the genetic software, PLINK version 1.07 [32]. SNPs were excluded from further analysis if they had a MAF of <5% or deviated from Hardy-Weinberg equilibrium (HWE) among control subjects (p < 0.001). We also excluded individuals with >20% of missing genotype data.

Genotyping data were then analyzed with unconditional logistic regression as implemented in PLINK to calculate odds ratios (ORs) and 95% confidence intervals (CIs) as risk estimates for colorectal cancer in subjects with a linear (log-odds additive) scoring for 0, 1, or 2 copies of the minor allele of each SNP. The analyzed models were adjusted for age (continuous) and hysterectomy status (yes, no) to reflect our case-control matching design. An empirical p value was calculated that gave a pointwise estimate of the significance level of each SNP; a value of <0.05 was denoted statistical significance.

Haplotype blocks were constructed for gene regions tagging ≥1 SNP with an allelic association test p value of <0.05. We first examined pairwise LD among controls and determined the LD blocks for these SNPs using the 'solid spine of LD' algorithm implemented in Haploview. Haplotype frequency and expected haplotypes for each subject were then inferred based on the unphased genotype data using the expectation-maximization (EM) algorithm in PLINK. We used unconditional logistic regression to estimate haplotype-specific ORs with the rest of the haplotypes as the referents. Haplotypes with estimated frequency of <1% were excluded from the analysis. We also performed an omnibus test to obtain a global p value for each haplotype block.

The possible joint effects of variation in each of the 8 genes on colorectal cancer risk were evaluated using the set-based tests implemented in PLINK. Within each gene, the set-based tests first selected the best SNP based on test statistic followed by SNPs in order of decreasing statistical significance. The statistic for each set (or each gene) with selected SNPs is calculated by averaging the test statistics from the selected SNPs within each gene. In the present analysis, we allowed the PLINK to choose simultaneously up to 5 independent loci (r^2^<0.5) with each p-value below 0.2 and tested for statistical significance with 10,000 permutations.

We also assessed effect modifiers including age (<70, ≥70 years), BMI (<25, ≥25 kg/m2), HT use (current E-alone users, current E+P users, past, never), and physical activity (<median, ≥median METs/wk) on the genetic association with colorectal cancer risk. We performed unconditional logistic regression analysis according to these factors with adjustment for the covariates described above. We also performed a global likelihood ratio test with a comparison of the log likelihood of the two models with and without the interaction terms in SAS version 9.0 (SAS Institute Inc, Cary, NC).

To control for comparisons for multiple SNPs (or haplotypes), we performed 10,000 permutations to generate a gene-specific (familywise) empirical p value for each SNP (or haplotype) and to determine how frequently the identified association would occur by chance. For each permutation, the case-control status was shuffled, and the maximum observed x^2 ^test statistic was compared with the experimental test statistics for each SNP (or haplotype). As compared with the asymptotic testing, the permutation procedures are not restricted to the assumptions of normality and Hardy-Weinberg equilibrium and are unaffected by rare allele frequency and small sample sizes. We performed these permutations using the max(T) permutation option in PLINK. In addition, we calculated the false discovery rate (FDR) for the global tests of interaction analysis on each gene using SAS procedure PROC MULTTEST [33].

In the present analysis, power to detect associated SNPs of a MAF of 10% with relative risks of 1.3, 1.4, 1.5, and 1.7 would be 13%, 27%, 46%, and 80%, respectively, using a 2-sided test and a p value of 0.001.

## Results

Twenty-two of the 272 SNPs were first removed because of an MAF of <5%. An additional 8 SNPs were excluded because the distribution of the genotypes deviated from HWE among controls (p < 0.001). The remaining 242 SNPs were included for further analysis (Additional file 1, Table S1). We also excluded 78 individuals with >20% of missing genotype data, resulting in a total of 1298 women in the present analysis.

Table 2 provides the comparison of baseline characteristics of colorectal cancer patients and control subjects. Compared with control subjects, cancer patients tended to be heavier, physically inactive, consumed fewer calories, and were less likely to receive screening exams. However, difference in distribution was not statistically significant between cases and controls with respect to current smoking, history of colon polyps, family history of colorectal cancer, alcohol intake, and current use of E-alone or E+P therapy.

**Table 2:** Baseline characteristics (mean or %) among colorectal cancer cases and control subjects of Caucasians from the Women's Health Initiative Observational Cohort

Characteristics	Cases (N = 427)	Controls (N = 871)	***P***_***value***_
Age, year	66.6	66.6	Matched
Body mass index, kg/m^2^	27.9	26.9	0.001
Family history of colorectal cancer, %	18.7	14.8	0.07
Colonoscopy/sigmoidoscopy exam, %	50.0	57.6	0.01
History of colorectal polyps, %	20.0	19.5	0.89
Hysterectomy, %	35.4	34.9	Matched
Current smoking, %	11.2	10.6	0.82
Current use of E-alone therapy, %	19.7	24.0	0.06
Current use of E+P therapy, %	15.7	21.2	0.06
Physical activity, MET/week	12.4	14.4	0.009
Alcohol intake, drink/day			0.07
never/past	22.2	26.0	
<1	38.9	30.8	
≥1	38.9	43.2	
Total calorie intake, g/day	1437	1556	0.001
Tumor location			
colon, N	338	N/A	
rectum, N	58	N/A	
Tumor stage			
Localized, N	183	N/A	
Regional, N	152	N/A	
Distant, N	61	N/A	

Among the 242 SNPs evaluated, rs10883782 and rs17724534 in *CYP17A1*, rs9340837 in *ESR1*, and rs1902584 in *CYP19A1 *were associated with colorectal cancer risk with an empirical p value of <0.05 (Table 3). When multiple comparisons were accounted for, only the *CYP17A1 *rs17724534 variant remained marginally significant (OR per copy of the risk allele (A) = 1.39, 95% CI = 1.08-1.78, corrected p-value = 0.07). There was no interaction between gene variants and BMI, types of HT, age, and physical activity in relation to colorectal cancer risk (corrected p values for interaction ≥0.14). There was also no association between gene variants and colorectal cancer risk according to tumor location and stage (data not shown).

**Table 3:** Variation in sex-steroid hormone synthesizing enzymes and receptor in relation to colorectal cancer risk in the Women's Health Initiative Observational Cohort

Gene/Variant	N total (case/control)	**MAF %**^**1 **^**(case/control)**	***OR (95% CI)***^**2**^	***P***_***trend***_^**3**^	***corrected P***_***trend***_^**4**^
**CYP17A1**					
rs10883782	410/834	15/18	0.78 (0.61-0.98)	0.03	0.22
rs17724534	406/829	15/12	**1.39 (1.08-1.78)**	0.009	0.07
**CYP19A1**					
rs1902584	410/811	6/5	1.45 (1.07-1.98)	0.02	0.36
**ESR1**					
rs9340837	424/866	12/9	1.37 (1.05-1.79)	0.02	0.73
					
**CYP17A1**					
rs10883782	47/102	17/13	1.40 (0.72-2.73)	0.32	0.92
rs17724534	50/103	1/4	0.22 (0.03-1.74)	0.12	0.72
**CYP19A1**					
rs1902584	52/108	6/7	0.79 (0.28-2.25)	0.67	0.99
**ESR1**					
rs9340837	52/107	46/46	1.02 (0.64-1.62)	0.95	0.99

^1^. MAF = minor allele frequency.

^2^.For each variant, odds ratios are per copy of the risk allele.

^3^. Pointwise 10,000 permutation tests.

^4^. Familywise 10,000 permutation tests.

Haplotype analysis was performed for the *CYP17A1, CYP19A1, and ESR1 *genes which included ≥1 SNP with an empirical p value of <0.05. The 5^th ^block of the *CYP19A1 *gene, which contains the promoter region with 8 tagging SNPs including rs1902584, was significantly associated with colorectal cancer risk (corrected global p value = 0.02). Specifically, the TCCGCCGT (OR = 0.51, 95% CI = 0.34-0.7) and ATCGCTGT (OR = 1.51, 95% CI = 1.13-2.01) haplotypes were associated with colorectal cancer risk (Additional file 2, Table S2). The LD blocks in *CYP17A1 *and *ESR1 *were not significantly associated with colorectal cancer risk (global p values ≥0.19).

We further evaluated the joint effects of independent loci on colorectal cancer risk. We found that the combined effects of rs10883782 and rs17724534 in *CYP17A1 *were significantly associated with colorectal cancer risk (empirical p value = 0.04). Around 24% of women carried ≥1 copy of the rs10883782 T allele and ≥1 copy of the rs17724534 A allele. Carriers who had both of these risk alleles were at a much greater risk of colorectal cancer (OR per copy of both risk alleles (T and A) = 4.60, 95% CI = 2.10-10.1). However, the LD for these 2 SNPs was low (r^2 ^= 3%).

We also examined whether findings of the 4 SNPs with pointwise significance in Caucasians might also be present in African Americans. The risk allele, A, of the *CYP17A1 *rs1772453 SNP was rare (MAF = 3%) in this ethnic group, which was not associated with colorectal cancer risk. Similarly, there was no association with the rest 3 SNPs in this ethnic group (p values ≥0.32) (Table 3).

## Discussion

In this study of common and coding variation in 3 sex hormone receptors (*ESR1, ESR2, PGR) *and 5 hormone-synthesizing enzymes (*CYP17A1, CYP19A1, HSD17B1, HSD17B2, HSD17B4*) in relation to colorectal cancer risk among WHI-OS women of European ancestry, we observed suggestive evidence for an association with the rs17724534 SNP in *CYP17A1*. The association with colorectal cancer was more pronounced when the risk attributable to this SNP was combined with that of another SNP (rs10883782) in the same gene. In addition, an LD block in *CYP19A1 *which includes the promoter region was significantly associated with risk for colorectal cancer. There was, however, little evidence for an association of variation in other genes with colorectal cancer risk. The overall genetic association was also not affected by modifying factors including HT use, BMI, physical activity, and age. Moreover, the null association seen in African Americans is not surprising given that power is also very limited in this group of women.

The biological activity of estrogen signaling in the colon remains unclear. Several rodent and cell line studies have shown that estrogen-activated signaling through estrogen receptor alpha and/or estrogen receptor beta exhibits growth inhibition effects on colon cancer cells and loss of either receptors has been detected in colorectal cancer [34-38]. It has also been suggested that estrogen (ie, estradiol) upregulates mismatch repair genes in colonic epithelium cells which coordinate the repair of nucleotide base mismatches [39,40]. However, other cell line studies have suggested the proliferative activity of estrogens in colon cancer cells [41,42]. A recent study of human colon carcinoma has also reported that local synthesized concentrations of estrogens were 2-fold higher in colon carcinoma than those in adjacent normal colonic mucosa and were associated with adverse clinical outcome of the patients [43]. These observations suggest that estrogens may have dual effects on colorectal cancer development.

Little is known about the mechanism by which progesterone prevents colorectal carcinogenesis. It has been shown in cell lines that administration with progesterone at high concentrations resulted in inhibition of colon cancer growth [38]. In addition, a lower expression of PGR has been reported in tumors than in normal colorectal mucosa [44]. Progesterone may also enhance the estrogenic effects on cells [45] and inhibit the mitogenic activity of *IGFs*, possibly through the regulation of *IGFBP1 *[46-48].

Few candidate-gene studies have evaluated variation in hormone receptors (*ESR1, ESR2, PGR*) in relation to colorectal cancer risk [9-12]. Two studies have reported an association with *ESR1 *(rs9340799) and *ESR2 *(rs1255953) variants [9,10], whereas our study along with two other studies [11,12] did not observe such an association. As common variants likely confer a small risk for disease development [21,22], studies with larger sample sizes than the current study may be required for the detection of significant association between sex hormone receptor genes and colorectal cancer [49]. In addition, our study may be underpowered to observe an association with variants in key enzymes involved in the formation of active estrogens (17beta-HSD families). Future large consortium studies may help shed light on the relationship between these gene variants and colorectal cancer.

The *CYP17A1 *gene encodes cytocrome P450C17alpha, an enzyme with 17 alpha-hydroxylase and 17,20-lyase activities at key points in the biosynthesis of androgens and estrogens via progesterone [50]. A change of T→C variant (rs743572) located in the *CYP17A1 *promoter region has been found to create a SP1-type (CCACC) promoter site [51] and the C allele is associated with increased CYP17 expression levels [52,53]. It has further been suggested that the *CYP17A1 *C allele is associated with enhanced production of all steroid hormones including progesterone and estrogen because of increased steroidogenesis in premenopausal women [54,55], although the association is much weaker in postmenopausal women [56]. In agreement with a previous case-control study of middle-aged men and women [11], we did not find an association between this SNP and colorectal cancer risk. However, rs17724534, a neighboring SNP which is in LD with rs743572, was found to be suggestively associated with colorectal cancer risk. In this study population, all women who carried the rs17724534 A allele also had T allele of rs743572, suggesting that the rs17724534 A allele may be associated with lower progesterone and estrogen levels, leading to an increased risk for colorectal cancer.

We also observed a significant interaction between rs17724534 and rs10883782 in the *CYP17A1 *gene on colorectal cancer risk. The A allele of rs17724534 paired with T allele of rs10883782 were associated with a much greater risk for colorectal cancer as compared with other alleles. It is possible that the rs10883782 T allele enhanced the risk associated with the rs17724534 A allele on colorectal cancer development. To date, the functional relevance of rs10883782 to colorectal cancer risk is unknown, although it has been suggested that rs10883782 is near or within a region showing sequence homology to a CCAAT/enhancer protein, which is known to be a strong transcription regulator [57]. Both SNPs, which are 21.6k bp apart and not in LD, belong to 2 discrete haplotype blocks in this study population.

After menopause, estrogen biosynthesis takes place predominantly in adipose tissue and is catalyzed by the aromatase enzyme, encoded by the *CYP19A1 *gene, which converts androgens to estrogens. In the present study, a haplotype block (block #5 in our analysis) in *CYP19A1 *was associated with colorectal cancer, with 2 haplotypes reaching pointwise significance levels. The risk estimates in one haplotype (ATCGCTGT) were similar to those from the tagging SNP (rs1902584) in this block with the minor allele (A) showing an elevated risk for colorectal cancer, suggesting that the observed association with this haplotype block likely reflects that with rs1902584. The rs1902584 SNP is near the promoter 1.4 region that regulates the transcription of the aromatase gene [24,58,59], thereby affecting circulating hormone levels. It has been suggested that the minor allele genotypes (AT or AA) are associated with higher estrogen levels as compared with the homozygous major allele genotype (TT) in overweight postmenopausal women [60], suggesting a potential link between the minor allele (A) and elevated estrogens from the adiposity. It is possible that our finding of the associated increased risk for colorectal cancer with the minor allele may be attributable to obesity-induced elevation of estrogen levels. We, however, observed no effect modification by BMI on the association with this SNP, likely due to a lack of power.

There are several limitations of the present study. First, the genotyped SNPs may not sufficiently cover the entire gene regions. Although we have chosen a commonly used selection threshold (r^2 ^≥70%) for tagging SNP selection and have also included several functional SNPs, we cannot rule out the possibility that other untyped variants may contribute to the risk of developing colorectal cancer. In addition, the current data focused only on common SNPs without assessing the potential contributions of rare variants. However, if rare variants are to be discovered with an increase in sample size, it is possible that unidentified variants will have increasingly small effects [61]. Moreover, we may have limited statistical power for analysis of most of our candidate genes. Power is also limited in this study for subgroup analysis according to potential risk modifiers and tumor characteristics. Finally, we do not have information on which part of the European regions (eg, south vs. north) our samples were from, which may potentially confound the findings.

## Conclusion

We observed little support for an association of gene variants in hormone receptors (*ESR1, ESR2, PGR*) and active estrogen synthesizers (*HSD17B1, HSD17B2, and HSD17B4*) with colorectal cancer risk among postmenopausal women of European descent. However, there is a suggestive evidence for an association with variation in *CYP17A1 *and *CYP19A1*. Our findings warrant confirmation in future studies.

## Competing interests

The authors declare that they have no competing interests.

## Authors' contributions

JHL carried out the genetic analysis and drafted the manuscript. SMZ acquired the data. JHL, SMZ, JEM, BBC, and RTC participated in the design of the study. SMZ, PK, JEM, and MJG participated in analyses and interpretation of data. All authors have helped to draft the manuscript, read, and approved the final manuscript.

## Pre-publication history

The pre-publication history for this paper can be accessed here:

http://www.biomedcentral.com/1471-2350/12/78/prepub

## Supplementary Material

Additional file 1 **Characteristics of the 272 SNPs**. Information on SNPs including gene name, location, alleles, and whether being included in the analysis. Click here for file

Additional file 2 **Haplotype-based association of CYP19A1 with colorectal cancer risk among the Caucasians from the Women's Health Initiative-Observational Cohort**. Results of haplotype analysis for the CYP19A1 gene. Click here for file
