# Supplementary material for: Estrogen and progesterone-related gene variants and colorectal cancer risk in women
Source: BMC Med Genet. 2011 May 31;12:78. doi: 10.1186/1471-2350-12-78 (PMC3125237; doi:10.1186/1471-2350-12-78)
Supplement: Additional file 1 — Characteristics of the 272 SNPs. Information on SNPs including gene name, location, alleles, and whether being included in the analysis. [file 1471-2350-12-78-S1.DOC]

**Table S1- Characteristics of the 272 SNPs.**

| Gene | Rsname | Chr^1^ | Position^2^ | a1 | a2 | Excluded from the analysis? |
| --- | --- | --- | --- | --- | --- | --- |
| HSD17B4 | rs11539471 | 5 | 118888837 | 0 | T | Exclude--MAF<5% |
| HSD17B4 | rs11205 | 5 | 118889612 | G | A |  |
| HSD17B4 | rs154632 | 5 | 118797197 | A | C |  |
| HSD17B4 | rs384346 | 5 | 118797373 | T | A |  |
| HSD17B4 | rs32646 | 5 | 118810629 | C | T |  |
| HSD17B4 | rs257977 | 5 | 118830120 | G | T | Exclude--MAF<5% |
| HSD17B4 | rs7706359 | 5 | 118836450 | G | A |  |
| HSD17B4 | rs25640 | 5 | 118839432 | A | G |  |
| HSD17B4 | rs2455463 | 5 | 118847574 | C | G |  |
| HSD17B4 | rs17453681 | 5 | 118872179 | G | C | Exclude--MAF<5% |
| HSD17B4 | rs32665 | 5 | 118875039 | T | C | Exclude--not in HWE |
| ESR1 | rs2348078 | 6 | 152397880 | G | A |  |
| ESR1 | rs2982709 | 6 | 152398437 | C | G |  |
| ESR1 | rs2982712 | 6 | 152399872 | C | T |  |
| ESR1 | rs3020364 | 6 | 152408811 | G | A |  |
| ESR1 | rs6901451 | 6 | 152412629 | A | G |  |
| ESR1 | rs3020368 | 6 | 152412883 | T | C |  |
| ESR1 | rs6930355 | 6 | 152413084 | C | T |  |
| ESR1 | rs9341016 | 6 | 152423691 | C | T |  |
| ESR1 | rs2273207 | 6 | 152424018 | G | A |  |
| ESR1 | rs9341019 | 6 | 152424381 | C | A |  |
| ESR1 | rs7772579 | 6 | 152084195 | C | A |  |
| ESR1 | rs3020372 | 6 | 152426621 | C | T |  |
| ESR1 | rs3778081 | 6 | 152428321 | G | A |  |
| ESR1 | rs6919225 | 6 | 152428583 | C | T |  |
| ESR1 | rs3798573 | 6 | 152431055 | G | A |  |
| ESR1 | rs2982894 | 6 | 152433735 | C | T | Exclude--not in HWE |
| ESR1 | rs12199102 | 6 | 152434254 | A | C |  |
| ESR1 | rs3798576 | 6 | 152438279 | C | T |  |
| ESR1 | rs2982896 | 6 | 152441186 | T | C |  |
| ESR1 | rs2474148 | 6 | 152454597 | T | G |  |
| ESR1 | rs2982900 | 6 | 152456685 | T | C |  |
| ESR1 | rs1999805 | 6 | 152110057 | G | A |  |
| ESR1 | rs3020383 | 6 | 152458472 | C | G |  |
| ESR1 | rs3778099 | 6 | 152460268 | C | T |  |
| ESR1 | rs3020384 | 6 | 152460883 | G | C |  |
| ESR1 | rs9341066 | 6 | 152461219 | A | G |  |
| ESR1 | rs3798577 | 6 | 152462823 | C | T |  |
| ESR1 | rs1062577 | 6 | 152465598 | A | T |  |
| ESR1 | rs2813543 | 6 | 152466171 | A | G |  |
| ESR1 | rs2813544 | 6 | 152467275 | G | A |  |
| ESR1 | rs1543403 | 6 | 152470397 | G | C |  |
| ESR1 | rs910416 | 6 | 152474595 | C | T |  |
| ESR1 | rs1361024 | 6 | 152112621 | A | G |  |
| ESR1 | rs9383963 | 6 | 152477851 | G | A |  |
| ESR1 | rs1336981 | 6 | 152124062 | C | T | Exclude--not in HWE |
| ESR1 | rs2504070 | 6 | 152126666 | A | G |  |
| ESR1 | rs2504069 | 6 | 152127210 | C | T |  |
| ESR1 | rs7742074 | 6 | 152129848 | A | G |  |
| ESR1 | rs2504063 | 6 | 152132400 | A | G |  |
| ESR1 | rs7767143 | 6 | 152137387 | G | A |  |
| ESR1 | rs2504066 | 6 | 152137522 | C | G |  |
| ESR1 | rs4242278 | 6 | 151999305 | A | G |  |
| ESR1 | rs1285058 | 6 | 152150732 | C | G |  |
| ESR1 | rs543650 | 6 | 152152636 | T | G |  |
| ESR1 | rs9478243 | 6 | 152159151 | A | G |  |
| ESR1 | rs2881766 | 6 | 152160812 | G | T |  |
| ESR1 | rs1285060 | 6 | 152161042 | 0 | G | Exclude--MAF<5% |
| ESR1 | rs11964281 | 6 | 152163135 | T | C |  |
| ESR1 | rs6903180 | 6 | 152166924 | A | G | Exclude--MAF<5% |
| ESR1 | rs488133 | 6 | 152167137 | T | C |  |
| ESR1 | rs2077647 | 6 | 152170770 | C | T |  |
| ESR1 | rs10484922 | 6 | 152174010 | T | C |  |
| ESR1 | rs851974 | 6 | 152013380 | G | A |  |
| ESR1 | rs3853248 | 6 | 152181579 | C | T |  |
| ESR1 | rs3844508 | 6 | 152181735 | G | A |  |
| ESR1 | rs9340789 | 6 | 152185581 | C | G |  |
| ESR1 | rs6926750 | 6 | 152194681 | G | A |  |
| ESR1 | rs6903763 | 6 | 152194849 | A | G | Exclude--MAF<5% |
| ESR1 | rs827421 | 6 | 152198815 | G | A |  |
| ESR1 | rs3853251 | 6 | 152202449 | G | A |  |
| ESR1 | rs2234693 | 6 | 152205028 | C | T |  |
| ESR1 | rs4870057 | 6 | 152213591 | G | A |  |
| ESR1 | rs712221 | 6 | 152221934 | T | A |  |
| ESR1 | rs851969 | 6 | 152020941 | G | A |  |
| ESR1 | rs1643821 | 6 | 152225244 | A | G |  |
| ESR1 | rs2431260 | 6 | 152234024 | G | C |  |
| ESR1 | rs1033181 | 6 | 152236514 | A | G |  |
| ESR1 | rs2175898 | 6 | 152238645 | C | T |  |
| ESR1 | HCV11414947 | 6 | 152239321 | T | C |  |
| ESR1 | rs4869747 | 6 | 152239804 | C | A |  |
| ESR1 | rs11155819 | 6 | 152241052 | C | T |  |
| ESR1 | rs9478249 | 6 | 152241124 | G | T |  |
| ESR1 | rs9322334 | 6 | 152241734 | A | G |  |
| ESR1 | rs9340837 | 6 | 152242056 | G | C |  |
| ESR1 | rs866457 | 6 | 152024106 | T | C |  |
| ESR1 | rs9322336 | 6 | 152242123 | C | T |  |
| ESR1 | rs6557168 | 6 | 152242894 | C | T |  |
| ESR1 | rs9322340 | 6 | 152244191 | T | C |  |
| ESR1 | rs9340851 | 6 | 152244871 | C | T |  |
| ESR1 | rs9478251 | 6 | 152244972 | G | C | Exclude--MAF<5% |
| ESR1 | rs11155820 | 6 | 152245903 | G | A |  |
| ESR1 | rs1606678 | 6 | 152251508 | T | C |  |
| ESR1 | rs4870059 | 6 | 152265225 | G | A | Exclude--MAF<5% |
| ESR1 | rs1514347 | 6 | 152271138 | T | C |  |
| ESR1 | rs2347867 | 6 | 152271543 | G | A |  |
| ESR1 | rs1293944 | 6 | 152039239 | A | G |  |
| ESR1 | rs988328 | 6 | 152282843 | C | T |  |
| ESR1 | rs1913476 | 6 | 152287409 | C | T |  |
| ESR1 | rs6912184 | 6 | 152301899 | G | A |  |
| ESR1 | rs4583998 | 6 | 152302361 | A | G |  |
| ESR1 | rs9371564 | 6 | 152303806 | A | G |  |
| ESR1 | rs1801132 | 6 | 152307215 | G | C |  |
| ESR1 | rs3003917 | 6 | 152308161 | G | A |  |
| ESR1 | rs6914211 | 6 | 152310233 | A | T |  |
| ESR1 | rs3020373 | 6 | 152313533 | T | C |  |
| ESR1 | rs3020377 | 6 | 152314091 | G | A |  |
| ESR1 | rs980280 | 6 | 152040416 | A | G |  |
| ESR1 | rs3020391 | 6 | 152318616 | G | A |  |
| ESR1 | rs3003920 | 6 | 152320011 | T | C |  |
| ESR1 | rs3020394 | 6 | 152320906 | G | A |  |
| ESR1 | rs3020395 | 6 | 152321497 | G | A |  |
| ESR1 | rs985192 | 6 | 152325171 | A | C |  |
| ESR1 | rs3003925 | 6 | 152326151 | G | A |  |
| ESR1 | rs985695 | 6 | 152328398 | T | C |  |
| ESR1 | rs2347869 | 6 | 152328988 | C | A |  |
| ESR1 | rs1884050 | 6 | 152329088 | C | G |  |
| ESR1 | rs1884052 | 6 | 152333059 | G | C |  |
| ESR1 | rs851993 | 6 | 152047704 | G | A |  |
| ESR1 | rs1884053 | 6 | 152333159 | C | T |  |
| ESR1 | rs2347871 | 6 | 152334353 | G | A |  |
| ESR1 | rs3020403 | 6 | 152337408 | G | C |  |
| ESR1 | rs3020404 | 6 | 152338679 | G | A |  |
| ESR1 | rs726282 | 6 | 152344347 | A | C |  |
| ESR1 | rs726283 | 6 | 152344702 | G | A |  |
| ESR1 | rs9397463 | 6 | 152346021 | T | C |  |
| ESR1 | rs3020407 | 6 | 152348954 | G | A |  |
| ESR1 | rs2144025 | 6 | 152349399 | T | C |  |
| ESR1 | rs7743290 | 6 | 152350825 | G | T |  |
| ESR1 | rs851987 | 6 | 152049582 | A | G |  |
| ESR1 | rs12212176 | 6 | 152351700 | T | C |  |
| ESR1 | rs7754762 | 6 | 152353230 | A | T |  |
| ESR1 | rs28385619 | 6 | 152354567 | T | A |  |
| ESR1 | rs9340944 | 6 | 152355411 | A | G |  |
| ESR1 | rs722208 | 6 | 152364578 | G | A |  |
| ESR1 | rs722209 | 6 | 152364889 | T | C |  |
| ESR1 | rs9340955 | 6 | 152371894 | A | C | Exclude--MAF<5% |
| ESR1 | rs3020411 | 6 | 152385456 | G | A |  |
| ESR1 | rs9478265 | 6 | 152390594 | A | G | Exclude--MAF<5% |
| ESR1 | rs926778 | 6 | 152397475 | A | C |  |
| ESR1 | rs2982571 | 6 | 152054432 | T | A |  |
| CYP17A1 | rs7089422 | 10 | 104595318 | A | G |  |
| CYP17A1 | rs17724534 | 10 | 104595511 | A | G |  |
| CYP17A1 | rs284861 | 10 | 104562266 | A | G |  |
| CYP17A1 | rs619824 | 10 | 104571278 | T | G |  |
| CYP17A1 | rs10883782 | 10 | 104573922 | C | T |  |
| CYP17A1 | rs4919682 | 10 | 104574320 | A | G |  |
| CYP17A1 | rs284849 | 10 | 104581172 | A | C |  |
| CYP17A1 | rs1004467 | 10 | 104584497 | C | T |  |
| CYP17A1 | rs4919687 | 10 | 104585238 | T | C |  |
| CYP17A1 | rs743572 | 10 | 104587142 | C | T |  |
| CYP17A1 | rs2486758 | 10 | 104587470 | G | A |  |
| PGR | rs1042838 | 11 | 100438622 | T | G |  |
| PGR | rs1824128 | 11 | 100439041 | A | C | Exclude--not in HWE |
| PGR | rs660149 | 11 | 100439524 | C | G |  |
| PGR | rs495997 | 11 | 100440990 | C | T |  |
| PGR | rs11224579 | 11 | 100442270 | A | G |  |
| PGR | rs559700 | 11 | 100468719 | G | A |  |
| PGR | rs516693 | 11 | 100468953 | A | G |  |
| PGR | rs508533 | 11 | 100470449 | C | A |  |
| PGR | rs601040 | 11 | 100472762 | T | C | Exclude--not in HWE |
| PGR | rs572483 | 11 | 100472782 | G | A | Exclude--not in HWE |
| PGR | rs477794 | 11 | 100396881 | A | G |  |
| PGR | rs555653 | 11 | 100474755 | C | T |  |
| PGR | rs543215 | 11 | 100479243 | C | T |  |
| PGR | rs613120 | 11 | 100479488 | C | T |  |
| PGR | rs529359 | 11 | 100499056 | G | A |  |
| PGR | rs481775 | 11 | 100499885 | T | C |  |
| PGR | rs11571150 | 11 | 100503682 | T | G | Exclude--MAF<5% |
| PGR | rs10895068 | 11 | 100505424 | A | G |  |
| PGR | rs518162 | 11 | 100505711 | T | C |  |
| PGR | rs948516 | 11 | 100515143 | C | T |  |
| PGR | rs474320 | 11 | 100519759 | T | A |  |
| PGR | rs12223660 | 11 | 100396913 | 0 | A | Exclude--MAF<5% |
| PGR | rs568157 | 11 | 100529492 | G | A |  |
| PGR | rs481883 | 11 | 100405345 | C | G |  |
| PGR | rs1545611 | 11 | 100405976 | C | T |  |
| PGR | rs1870019 | 11 | 100406683 | C | T |  |
| PGR | rs1824125 | 11 | 100407263 | T | G |  |
| PGR | rs500760 | 11 | 100415201 | G | A |  |
| PGR | rs1042839 | 11 | 100427412 | T | C |  |
| PGR | rs545835 | 11 | 100437015 | T | C |  |
| ESR2 | rs1256049 | 14 | 63793804 | A | G | Exclude--MAF<5% |
| ESR2 | rs1273196 | 14 | 63809258 | C | T |  |
| ESR2 | rs1256031 | 14 | 63815932 | C | T |  |
| ESR2 | rs1271572 | 14 | 63831670 | T | G |  |
| ESR2 | rs2978381 | 14 | 63836405 | G | A |  |
| ESR2 | rs3020450 | 14 | 63838055 | A | G |  |
| ESR2 | rs1152590 | 14 | 63750946 | T | C |  |
| ESR2 | rs8020646 | 14 | 63761073 | G | A |  |
| ESR2 | rs928554 | 14 | 63763948 | C | T |  |
| ESR2 | rs4986938 | 14 | 63769569 | A | G |  |
| ESR2 | rs944050 | 14 | 63769798 | C | T | Exclude--MAF<5% |
| ESR2 | rs1256064 | 14 | 63770492 | G | A |  |
| ESR2 | rs1256063 | 14 | 63771970 | A | G |  |
| ESR2 | rs8017441 | 14 | 63785547 | C | T | Exclude--not in HWE |
| ESR2 | rs1256054 | 14 | 63786066 | 0 | C | Exclude--MAF<5% |
| CYP19A1 | rs4646 | 15 | 49290136 | T | G |  |
| CYP19A1 | rs10046 | 15 | 49290278 | C | T |  |
| CYP19A1 | rs17601241 | 15 | 49295166 | T | C |  |
| CYP19A1 | rs6493487 | 15 | 49301021 | C | T |  |
| CYP19A1 | rs28757184 | 15 | 49301864 | T | C | Exclude--MAF<5% |
| CYP19A1 | rs2899472 | 15 | 49303347 | T | G |  |
| CYP19A1 | rs12439137 | 15 | 49303596 | C | T |  |
| CYP19A1 | rs700518 | 15 | 49316404 | A | G |  |
| CYP19A1 | rs2414096 | 15 | 49317071 | C | T |  |
| CYP19A1 | rs10519295 | 15 | 49319939 | G | A |  |
| CYP19A1 | rs2899469 | 15 | 49269214 | G | A |  |
| CYP19A1 | rs10519296 | 15 | 49320386 | G | C |  |
| CYP19A1 | rs4775936 | 15 | 49323314 | A | G |  |
| CYP19A1 | rs10459592 | 15 | 49323433 | A | C |  |
| CYP19A1 | rs767199 | 15 | 49327679 | T | C |  |
| CYP19A1 | rs7172156 | 15 | 49333590 | T | C |  |
| CYP19A1 | rs1008805 | 15 | 49336891 | C | T |  |
| CYP19A1 | rs6493494 | 15 | 49337127 | T | C |  |
| CYP19A1 | rs749292 | 15 | 49346023 | T | C |  |
| CYP19A1 | rs11636639 | 15 | 49350384 | C | A |  |
| CYP19A1 | rs1902586 | 15 | 49358145 | T | C |  |
| CYP19A1 | rs8023699 | 15 | 49270526 | C | A |  |
| CYP19A1 | rs936306 | 15 | 49366890 | A | G |  |
| CYP19A1 | rs936307 | 15 | 49367267 | T | G |  |
| CYP19A1 | rs17523880 | 15 | 49379835 | T | G |  |
| CYP19A1 | rs2470152 | 15 | 49382264 | T | C |  |
| CYP19A1 | rs17523922 | 15 | 49386497 | C | G |  |
| CYP19A1 | rs2445759 | 15 | 49388133 | A | C |  |
| CYP19A1 | rs28566535 | 15 | 49388433 | C | A |  |
| CYP19A1 | rs3751592 | 15 | 49393870 | G | A |  |
| CYP19A1 | rs3751591 | 15 | 49394002 | C | T |  |
| CYP19A1 | rs1902584 | 15 | 49398946 | A | T |  |
| CYP19A1 | rs12594395 | 15 | 49270999 | G | C |  |
| CYP19A1 | rs1004984 | 15 | 49400821 | T | C |  |
| CYP19A1 | rs28757082 | 15 | 49403325 | A | C | Exclude--MAF<5% |
| CYP19A1 | rs8041933 | 15 | 49405038 | T | C |  |
| CYP19A1 | rs2470144 | 15 | 49409017 | G | A |  |
| CYP19A1 | rs7174997 | 15 | 49409420 | A | C |  |
| CYP19A1 | rs6493497 | 15 | 49418127 | T | C |  |
| CYP19A1 | rs2445765 | 15 | 49422190 | G | C |  |
| CYP19A1 | rs2446405 | 15 | 49434085 | T | A |  |
| CYP19A1 | rs8031580 | 15 | 49274962 | A | G |  |
| CYP19A1 | rs4775931 | 15 | 49277912 | A | T |  |
| CYP19A1 | rs9972359 | 15 | 49279146 | G | A |  |
| CYP19A1 | rs16964189 | 15 | 49281529 | A | G |  |
| CYP19A1 | rs934632 | 15 | 49283122 | T | C |  |
| CYP19A1 | rs4275794 | 15 | 49288409 | G | A |  |
| HSD17B2 | rs1364287 | 16 | 80676601 | A | G |  |
| HSD17B2 | rs8191225 | 16 | 80682500 | C | T |  |
| HSD17B2 | rs9934209 | 16 | 80684331 | C | G |  |
| HSD17B2 | rs2955163 | 16 | 80685698 | C | G | Exclude--MAF<5% |
| HSD17B2 | rs2955162 | 16 | 80686278 | T | C |  |
| HSD17B2 | rs996752 | 16 | 80690493 | G | A |  |
| HSD17B2 | rs4889459 | 16 | 80691579 | T | C |  |
| HSD17B2 | rs1364284 | 16 | 80693755 | A | G |  |
| HSD17B2 | rs4378606 | 16 | 80694249 | A | G |  |
| HSD17B2 | rs11649253 | 16 | 80694856 | G | A | Exclude--MAF<5% |
| HSD17B2 | rs6564958 | 16 | 80608616 | T | C |  |
| HSD17B2 | rs10514527 | 16 | 80695143 | C | A |  |
| HSD17B2 | rs9923501 | 16 | 80698062 | C | T |  |
| HSD17B2 | rs4404064 | 16 | 80611000 | C | T |  |
| HSD17B2 | rs4291899 | 16 | 80622339 | T | G |  |
| HSD17B2 | rs4445895 | 16 | 80626398 | T | C |  |
| HSD17B2 | rs4243229 | 16 | 80643099 | A | G | Exclude--MAF<5% |
| HSD17B2 | rs8191175 | 16 | 80662957 | C | G | Exclude--MAF<5% |
| HSD17B2 | rs2966245 | 16 | 80663800 | G | A |  |
| HSD17B2 | rs2042429 | 16 | 80670972 | C | T |  |
| HSD17B2 | rs7201637 | 16 | 80672776 | A | T | Exclude--MAF<5% |
| HSD17B1 | rs2071046 | 17 | 37943139 | G | C |  |
| HSD17B1 | rs607795 | 17 | 37952858 | T | C |  |
| HSD17B1 | rs2676530 | 17 | 37959481 | T | C |  |
| HSD17B1 | rs676387 | 17 | 37959799 | A | C |  |
| HSD17B1 | rs605059 | 17 | 37960432 | G | A |  |
| HSD17B1 | rs12602084 | 17 | 37965295 | T | C | Exclude--not in HWE |
| HSD17B1 | rs598126 | 17 | 37970046 | A | G |  |

^1^. Chr=chromosome.

^2^. Based on NCBI build 36.
