# Supplementary material for: Estrogen and progesterone-related gene variants and colorectal cancer risk in women
Source: BMC Med Genet. 2011 May 31;12:78. doi: 10.1186/1471-2350-12-78 (PMC3125237; doi:10.1186/1471-2350-12-78)
Supplement: Additional file 2 — Haplotype-based association of CYP19A1 with colorectal cancer risk among the Caucasians from the Women's Health Initiative-Observational Cohort. Results of haplotype analysis for the CYP19A1 gene. [file 1471-2350-12-78-S2.DOC]

**Table S2- Haplotype-based association of CYP19A1 with colorectal cancer risk among the Caucasians from the Women’s Health Initiative-Observational Cohort.**

| Gene/Block # | N case (%) | N control (%) | OR (95% CI) | *P_trend_** | *corrected*  *P_trend_*† |
| --- | --- | --- | --- | --- | --- |
| **Block 1** |  |  |  | 0.23 | 0.63 |
| GACAA | 239 (26.0) | 466 (2.51) | 1.05 (0.87-1.26) |  |  |
| ACGAT | 175 (19.0) | 355 (19.1) | 1.00 (0.82-1.22) |  |  |
| AAGGT | 166 (18.0) | 401 (21.6) | 1.04 (0.84-1.23) |  |  |
| GACGT | 160 (17.4) | 313 (16.9) | 0.80 (0.66-0.98) |  |  |
| AACGT | 169 (18.4) | 299 (16.1) | 1.18 (0.95-1.45) |  |  |
| **Block 2** |  |  |  | 0.04 | 0.17 |
| GCAGTCTT | 290 (27.0) | 607 (26.6) | 1.02 (0.87-1.20) |  |  |
| GTATCCTG | 90 (8.4) | 159 (7.0) | 1.22 (0.93-1.59) |  |  |
| GCAGTCTG | 245 (23.1) | 621 (27.2) | 0.80 (0.68-0.95) |  |  |
| GTATCCCG | 102 (9.5) | 202 (8.8) | 1.08 (0.84-1.38) |  |  |
| ACGGCCTG | 71 (6.6) | 113 (5.0) | 1.36 (1.00-1.84) |  |  |
| ACGGCCCG | 153 (14.2) | 347 (15.2) | 0.93 (0.75-1.14) |  |  |
| GCATCTTG | 78 (7.3) | 142 (6.2) | 1.18 (0.89-1.57) |  |  |
| **Block 3** |  |  |  | 0.06 | 0.23 |
| GTACACT | 245 (26.0) | 604 (30.8) | 0.79 (0.66-0.94) |  |  |
| GTGCACT | 96 (10.2) | 171 (8.7) | 1.19 (0.92-1.55) |  |  |
| ACACGAC | 357 (37.9) | 743 (37.8) | 1.00 (0.86-1.18) |  |  |
| GTAGACT | 56 (5.9) | 90 (4.6) | 1.31 (0.93-1.85) |  |  |
| GTACACC | 28 (3.0) | 61 (3.1) | 0.96 (0.61-1.51) |  |  |
| ACACGCC | 101 (10.7) | 169 (8.6) | 1.28 (0.99-1.66) |  |  |
| GTACGAT | 32 (3.4) | 63 (3.2) | 1.06 (0.69-1.64) |  |  |
| **Block 4** |  |  |  | 0.70 | 0.99 |
| CTTTCCGGGCGC | 281 (25.7) | 590 (26.1) | 0.98 (0.83-1.16) |  |  |
| CTTTCCGGTCGC | 159 (14.6) | 352 (15.6) | 0.92 (0.75-1.13) |  |  |
| CTCCACATGTGA | 54 (5.0) | 118 (5.2) | 0.95 (0.68-1.32) |  |  |
| TCCCACGGGTGC | 276 (25.3) | 567 (25.1) | 1.01 (0.86-1.19) |  |  |
| CTCCATAGGCGC | 38 (3.5) | 57 (2.5) | 1.40 (0.92-2.12) |  |  |
| CCCCACGGGTGC | 25 (2.3) | 63 (2.8) | 0.82 (0.51-1.30) |  |  |
| TCCCACGGGTCC | 120 (11.0) | 213 (9.4) | 1.19 (0.94-1.50) |  |  |
| CTCCACATGCGC | 19 (1.7) | 37 (1.6) | 1.07 (0.61-1.86) |  |  |
| TCCCACGGGCGC | 26 (2.4) | 61 (2.7) | 0.88 (0.55-1.39) |  |  |
| **Block 5** |  |  |  | 0.005 | 0.02 |
| TCCACCCA | 315 (32.3) | 623 (30.8) | 1.07 (0.91-1.27) |  |  |
| TTTGCCCA | 191 (19.6) | 426 (21.1) | 0.91 (0.75-1.11) |  |  |
| TCCAACCA | 187 (19.2) | 374 (18.5) | 1.05 (0.56-1.27) |  |  |
| ATCGCTGT | 85 (8.7) | 121 (6.0) | **1.50 (1.13-2.01)** |  |  |
| TCCGCCCA | 40 (4.1) | 105 (5.2) | 0.79 (0.54-1.14) |  |  |
| TCCGCCGT | 30 (3.1) | 119 (5.9) | **0.51 (0.34-0.77)** |  |  |
| TTCGCCCA | 51 (5.2) | 95 (4.7) | 1.13 (0.80-1.60) |  |  |
| TTCGCTGT | 35 (3.6) | 84 (4.3) | 0.84 (0.56-1.25) |  |  |

*. Pointwise 10,000 permutation tests.

†. Familywise 10,000 permutation tests.
